# Supplementary material for: Pluripotency factors determine gene expression repertoire at zygotic genome activation
Source: Nat Commun. 2022 Feb 10;13:788. doi: 10.1038/s41467-022-28434-1 (PMC8831532; doi:10.1038/s41467-022-28434-1)
Supplement: Supplementary file 3 — Description of Additional Supplementary Files [file 41467_2022_28434_MOESM3_ESM.docx]

**Description of Additional Supplementary Files**

Title: Supplementary Data 1

Description: Maternal and Zygotic Transcripts in the wild-type

Title: Supplementary Data 2

Description: Zygotic transcripts upregulated in the MZsox19b, MZspg and MZsox19bspg mutants

Title: Supplementary Data 3

Description: Accessible chromatin regions

Title: Supplementary Data 4

Description: Pou5f3, SoxB1 and Nanog peaks, consensus motifs used in this study

Title: Supplementary Data 5

Description: Table for Chi-squared test.

Title: Supplementary Movie 1

Description: Developmental of the wild-type and mutants: Part1: WT, MZsox19b and Msox19b Part2: WT, MZsox19b, MZspg, MZsox19bspg Part3: WT, MZspg, MZsox19bspg Part4: WT, Mspg, Msox19bspg Part5: WT, Msox19b, Msox19bspg

Title: Supplementary Movie 2

Description: Analysis of time series using RNAsense
