# Supplementary material for: Pluripotency factors determine gene expression repertoire at zygotic genome activation
Source: Nat Commun. 2022 Feb 10;13:788. doi: 10.1038/s41467-022-28434-1 (PMC8831532; doi:10.1038/s41467-022-28434-1)
Supplement: Supplementary file 11 — Reporting Summary [file 41467_2022_28434_MOESM11_ESM.pdf]

nature research

Corresponding author(s): Daria Onichtchouk
Last updated by author(s): Jan 14, 2021

Reporting Summary

Nature Research wishes to improve the reproducibility of the work that we publish. This form provides structure for consistency and transparency in reporting. For further information on Nature Research policies, see our Editorial Policies and the Editorial Policy Checklist.

Statistics

For all statistical analyses, confirm that the following items are present in the figure legend, table legend, main text, or Methods section.

n/a	Confirmed	
☐	☒	The exact sample size (*n*) for each experimental group/condition, given as a discrete number and unit of measurement
☐	☒	A statement on whether measurements were taken from distinct samples or whether the same sample was measured repeatedly
☐	☒	The statistical test(s) used AND whether they are one- or two-sided<br>*Only common tests should be described solely by name; describe more complex techniques in the Methods section.*
☐	☒	A description of all covariates tested
☐	☒	A description of any assumptions or corrections, such as tests of normality and adjustment for multiple comparisons
☐	☒	A full description of the statistical parameters including central tendency (e.g. means) or other basic estimates (e.g. regression coefficient) AND variation (e.g. standard deviation) or associated estimates of uncertainty (e.g. confidence intervals)
☐	☒	For null hypothesis testing, the test statistic (e.g. *F*, *t*, *r*) with confidence intervals, effect sizes, degrees of freedom and *P* value noted<br>*Give P values as exact values whenever suitable.*
☒	☐	For Bayesian analysis, information on the choice of priors and Markov chain Monte Carlo settings
☒	☐	For hierarchical and complex designs, identification of the appropriate level for tests and full reporting of outcomes
☒	☐	Estimates of effect sizes (e.g. Cohen's *d*, Pearson's *r*), indicating how they were calculated

Our web collection on statistics for biologists contains articles on many of the points above.

Software and code

Policy information about availability of computer code

Data collection: Illumina Casava1.7 software used for basecalling

Data analysis:
Adobe Photoshop CS4 (Adobe)
AxioVision SE64 Rel. 4.9.1 software (Carl Zeiss)
BamTools Barnett et al., 2011 in usegalaxy.eu
Bedtools Quinlan and Hall,2010 BEDTools in usegalaxy.eu
Bowtie2 Langmead and Salzberg, 2012 Bowtie2 in usegalaxy.eu
DAVID version 6.8 Huang da et al., 2009 in https://david.ncifcrf.gov/
DeepTools2 Ramirez et al., 2016 deepTools in usegalaxy.eu
DESeq2 Love et al., 2014 DESeq2 in usegalaxy.eu
European Galaxy server Afgan et al., 2018 https://usegalaxy.eu/
Feature count Liao et al., 2014 in usegalaxy.eu
FIMO Grant et al., 2011 in usegalaxy.eu
geecee program https://www.bioinformatics.nl/cgi-bin/emboss/geecee
GREAT: Genomic Regions Enrichment of Annotations Tool, version 3.0.0 Hiller et al., 2013 http://great.stanford.edu/great/public-3.0.0/html/
ImageJ versions 1.50i ,1.53a, 1.53c https://imagej.nih.gov/ij/download.html
JASPAR vertebrate non-redundant database Fornes et al., 2020 https://jaspar.genereg.net/
MEME suite Bailey and Gribskov, 1998 in usegalaxy.eu
Nucleosome prediction program Kaplan et al., 2009 and https://github.com/bgruening/galaxytools, Nucleosome Predictions in usegalaxy.eu
MACS2 Ferg et al., 2007 MACS2 callpeak in usegalaxy.eu
MEME suite Bailey and Gribskov, 1998 in usegalaxy.eu
RNA Star Dobin et al., 2013 RNA Star in usegalaxy.eu

RNA-sense this work https://bioconductor.org/packages/release/bioc/html/RNAsense.html
Tomtom Gupta et al., 2007 at https://meme-suite.org/meme/tools/tomtom
Trimmomatics Bolger et al., 2014 in usegalaxy.eu
UCSC browser at https://genome.ucsc.edu/
Zen2 software (Carl Zeiss)

R packages
NBPseq:
https://cran.rstudio.com/web/packages/NBPSeq/index.html
corrplot:
Wei T, Simko V (2021). R package 'corrplot': Visualization of a Correlation Matrix. (Version 0.92), https://github.com/taiyun/corrplot
ggplot2 :
H. Wickham. ggplot2: Elegant Graphics for Data Analysis. Springer-Verlag New York, 2016.
dplyr:
Hadley Wickham, Romain FranÁois, Lionel Henry and Kirill M¸ller (2020). dplyr: A Grammar of Data Manipulation. R package version 1.0.2. https://CRAN.R-project.org/package=dplyr
eulerr:
Larsson J (2020). _eulerr: Area-Proportional Euler and Venn Diagrams with Ellipses_. R package version 6.1.0, <URL: https://cran.r-project.org/package=eulerr>.
NBPSeq:
Yanming Di, Daniel W Schafer, with contributions from Jason S Cumbie and Jeff H Chang. (2014). NBPSeq: Negative Binomial Models for RNA-Sequencing Data. R package version 0.3.0. https://CRAN.R-project.org/package=NBPSeq
qvalue:
John D. Storey, Andrew J. Bass, Alan Dabney and David Robinson (2019). qvalue: Q-value estimation for false discovery rate control. R package version 2.16.0. http://github.com/jdstorey/qvalue
SummarizedExperiment:
Martin Morgan, Valerie Obenchain, Jim Hester and HervÈ PagËs (2019). SummarizedExperiment: SummarizedExperiment container. R package version 1.14.1.
reshape2:
Hadley Wickham (2007). Reshaping Data with the reshape Package. Journal of Statistical Software, 21(12), 1-20. URL http://www.jstatsoft.org/v21/i12/.
tidyverse:
Wickham et al., (2019). Welcome to the tidyverse. Journal of Open Source Software, 4(43), 1686, https://doi.org/10.21105/joss.01686
matrixStats:
Henrik Bengtsson (2020). matrixStats: Functions that Apply to Rows and Columns of Matrices (and to Vectors). R package version 0.57.0. https://CRAN.R-project.org/package=matrixStats
hrbrthemes:
Bob Rudis (2020). hrbrthemes: Additional Themes, Theme Components and Utilities for 'ggplot2'. R package version 0.8.0. https://CRAN.R-project.org/package=hrbrthemes
viridis:
Simon Garnier (2018). viridis: Default Color Maps from 'matplotlib'. R package version 0.5.1. https://CRAN.R-project.org/package=viridis
parallel:
R Core Team (2019). R: A language and environment for statistical computing. R Foundation for Statistical Computing, Vienna, Austria. URL https://www.R-project.org/.
stats:
R Core Team (2019). R: A language and environment for statistical computing. R Foundation for Statistical Computing, Vienna, Austria. URL https://www.R-project.org/.
forcats:
Hadley Wickham (2020). forcats: Tools for Working with Categorical Variables (Factors). R package version 0.5.0. https://CRAN.R-project.org/package=forcats

For manuscripts utilizing custom algorithms or software that are central to the research but not yet described in published literature, software must be made available to editors and reviewers. We strongly encourage code deposition in a community repository (e.g. GitHub). See the Nature Research guidelines for submitting code & software for further information.

Data

Policy information about availability of data

All manuscripts must include a data availability statement. This statement should provide the following information, where applicable:
- Accession codes, unique identifiers, or web links for publicly available datasets
- A list of figures that have associated raw data
- A description of any restrictions on data availability

The RNA-seq data generated in this study have been deposited in the GEO database under accession code GSE137424 [https://www.ncbi.nlm.nih.gov/geo/query/acc.cgi?acc=GSE137424]. The ATAC-seq data generated in this study have been deposited in the GEO database under accession code GSE188364 [https://www.ncbi.nlm.nih.gov/geo/query/acc.cgi?acc=GSE188364]. The H3K27ac and H3K4me3 ChIP-seq data generated in this study have been deposited in the GEO database under accession code GSE143306 [https://www.ncbi.nlm.nih.gov/geo/query/acc.cgi?acc=GSE143306]. The MZsox19b MNase-seq data generated in this

study have been deposited in the GEO database under accession code GSE125945 [https://www.ncbi.nlm.nih.gov/geo/query/acc.cgi?acc=GSE125945]. The WT and MZspg MNase-seq data used in this study are available in the GEO database under accession code GSE109410 [https://www.ncbi.nlm.nih.gov/geo/query/acc.cgi?acc=GSE109410]. The ChIP-seq data for Pou5f3, SoxB1 and Nanog TF binding used in this study are available in the GEO database under accession codes GSE39780 [https://www.ncbi.nlm.nih.gov/geo/query/acc.cgi?acc=GSE39780] and GSE34683 [https://www.ncbi.nlm.nih.gov/geo/query/acc.cgi?acc=GSE34683].The source data underlying main Fig.3 b,d,e, Fig.4 a,c, Supplementary Fig. S3 a, Fig. S4a,c, and S5 are provided as a Source Data file. The source data underlying main Fig.3 b,c,d, Supplementary Fig. S4b and S5 are provided as a Dataset S1. The source data underlying main Fig.4 a,b are provided as a Dataset S2. The source data underlying main Fig.5 a,b,d,e,f,g, Fig.6 b,c,e,f, Fig.7 a,b,c,e, Fig.8a,b, Supplementary Fig. S6, S7, S8a-e and S9 are provided as a Dataset S3. The source data underlying main Fig.5 e,f, Fig.6 a,d, Fig.7c, Supplementary Fig.S7 and S8b are provided as a Dataset S4.The source data underlying main Fig.5 c, Fig.7d, Supplementary Fig. S8 f,g are provided as a Dataset S5.

Field-specific reporting

Please select the one below that is the best fit for your research. If you are not sure, read the appropriate sections before making your selection.

- [x] Life sciences
- [ ] Behavioural & social sciences
- [ ] Ecological, evolutionary & environmental sciences

For a reference copy of the document with all sections, see nature.com/documents/nr-reporting-summary-flat.pdf

Life sciences study design

All studies must disclose on these points even when the disclosure is negative.

Sample size

Sample sizes for RNA-seq, ChIP-seq, MNase-seq and ATAC-seq were selected based on the common practice in the field. No sample size calculation was performed. i.e. similar sample sizes were used in zebrafish for transcriptome analysis and RNA-seq in (Aanes et al., 2014,Onichtchouk et al., 2010,White et al., 2017), for ATAC-seq (Miao et al., 2020; Palfy et al., 2020), for ChIP-seq in (Murphy et al., 2018; Vastenhouw et al., 2019), for MNase-seq in(Veil et al., 2019; Zhang et al., 2014)

Aanes, H., Collas, P., Alestrom, P., 2014. Transcriptome dynamics and diversity in the early zebrafish embryo. Briefings in functional genomics 13, 95-105.
Miao, L., Tang, Y., Bonneau, A.R., Chan, S.H., Kojima, M.L., Pownall, M.E., Vejnar, C.E., Giraldez, A.J., 2020. Synergistic activity of Nanog, Pou5f3, and Sox19b establishes chromatin accessibility and developmental competence in a context-dependent manner. bioRxiv, 2020.2009.2001.278796.
Murphy, P.J., Wu, S.F., James, C.R., Wike, C.L., Cairns, B.R., 2018. Placeholder Nucleosomes Underlie Germline-to-Embryo DNA Methylation Reprogramming. Cell 172, 993-1006 e1013.
Onichtchouk, D., Geier, F., Polok, B., Messerschmidt, D.M., Mossner, R., Wendik, B., Song, S., Taylor, V., Timmer, J., Driever, W., 2010. Zebrafish Pou5f1-dependent transcriptional networks in temporal control of early development. Mol Syst Biol 6, 354.
Palfy, M., Schulze, G., Valen, E., Vastenhouw, N.L., 2020. Chromatin accessibility established by Pou5f3, Sox19b and Nanog primes genes for activity during zebrafish genome activation. PLoS Genet 16, e1008546.
Vastenhouw, N.L., Cao, W.X., Lipshitz, H.D., 2019. The maternal-to-zygotic transition revisited. Development 146.
Veil, M., Yampolsky, L., Gruening, B., Onichtchouk, D., 2019. Pou5f3, SoxB1, and Nanog remodel chromatin on High Nucleosome Affinity Regions at Zygotic Genome Activation. Genome Res.
White, R.J., Collins, J.E., Sealy, I.M., Wali, N., Dooley, C.M., Digby, Z., Stemple, D.L., Murphy, D.N., Billis, K., Hourlier, T., Fullgrabe, A., Davis, M.P., Enright, A.J., Busch-Nentwich, E.M., 2017. A high-resolution mRNA expression time course of embryonic development in zebrafish. Elife 6.
Zhang, Y., Vastenhouw, N.L., Feng, J., Fu, K., Wang, C., Ge, Y., Pauli, A., van Hummelen, P., Schier, A.F., Liu, X.S., 2014. Canonical nucleosome organization at promoters forms during genome activation. Genome Res 24, 260-266.

Data exclusions

There were no data exclusions

Replication

RNA-seq experiments were performed at 2 to 4 biological replicates. ATAC-seq experiments were performed at 2 to 4 biological replicates. For ChIP-seq in two out of three genotypes (WT and MZspg), biological replicates were pooled before the library preparation, to achieve sufficient amount of starting material. For one genotype (MZsox19b), two libraries were from biological duplicates were prepared. To provide justification for experimental quality, we divided the whole zebrafish genome into 1 kb bins, and computed Pairwise Pearson correlations and Principle Component Analysis (PCA) between four libraries (Methods). The difference between the biological replicates was negligible when compared to the difference between the genotypes (Fig. S8a,b), which demonstrated the reproducibility of our ChIP-seq protocol and validated the use of single replicates for our purposes. For MNase-seq experiment in MZsox19b one biological replicate was performed. We confirm that all attempts of replication were successful for all experiments mentioned above.

Randomization

The samples were allocated into experiemntal groups by genotype (wild-type, MZsox19b, MZspg, MZsox19bspg)

Blinding

Blinding was not relevant for our study, because the mutant phenotypes are clearly distinguishable from each other and from the wild-type

Reporting for specific materials, systems and methods

We require information from authors about some types of materials, experimental systems and methods used in many studies. Here, indicate whether each material, system or method listed is relevant to your study. If you are not sure if a list item applies to your research, read the appropriate section before selecting a response.

Materials & experimental systems

n/a	Involved in the study
☐	☒ Antibodies
☒	☐ Eukaryotic cell lines
☒	☐ Palaeontology and archaeology
☐	☒ Animals and other organisms
☒	☐ Human research participants
☒	☐ Clinical data
☒	☐ Dual use research of concern

Methods

n/a	Involved in the study
☐	☒ ChIP-seq
☒	☐ Flow cytometry
☒	☐ MRI-based neuroimaging

Antibodies

Antibodies used

Anti-Histone H3 (acetyl K27) rabbit, 1/100 dilution Abcam plc., Cambridge, UK ab 4729
Anti-Histone H3 (tri-methyl K4) rabbit, 1/100 dilution Millipore Co., Temecula, California, USA 07-449

Validation

Real time PCR quality control was performed as described in the Methods section of the manuscript, "ChIP quality control and library preparation for histone marks" . Namely, To estimate the signal to background ratio in each ChIP experiment, we have chosen the positive and negative reference genomic regions, enriched in or devoid of chromatin marks. According to previously published data, the chromatin region near tiparp gene was highly enriched in H3K27ac and H3K4me3 histone marks at 4.3 hpf, while genomic region near igsf2 gene was not enriched in any of these marks. We performed quantitative PCR in ChIP and Input control material, using the primers for these regions. PCR primers used were: tiparp_f_1 5' CGCTCCCAACTCCATGTATC-3', tiparp_r_1 5'-AACGCAAGCCAAACGATCTC-3',igsf2_f_2 5'-GAACTGCATTAGAGACCCAC-3', igsf2_r_2 5'-CAATCAACTGGGAAAGCATGA-3'. QPCR was carried out using the SsoAdvanced™ Universal SYBR® Green Supermix from BIO-RAD. ChIP and input were normalized by ddCT method, using negative reference region (igsf2). The ChIP experiment was considered successful, if the enrichment in ChIP over input control on the positive reference region (tiparp) was more than 5-fold.

Animals and other organisms

Policy information about studies involving animals; ARRIVE guidelines recommended for reporting animal research

Laboratory animals

Danio rerio (zebrafish), males and females 0-2 xears old, strains: AB/TL, MZspg, MZsox19b, MZsox19bspg

Wild animals

no wild animals were used

Field-collected samples

no samples collected in the field were used

Ethics oversight

All experiments were performed in accordance with German Animal Protection Law (TierSchG) and European Convention on the Protection of Vertebrate Animals Used for Experimental and Other Scientific Purposes (Strasburg, 1986). The generation of double mutants was approved by the Ethics Committee for Animal Research of the Koltzov Institute of Developmental Biology RAS, protocol 26 from 14.02.2019

Note that full information on the approval of the study protocol must also be provided in the manuscript.

ChIP-seq

Data deposition

☒ Confirm that both raw and final processed data have been deposited in a public database such as GEO.

☒ Confirm that you have deposited or provided access to graph files (e.g. BED files) for the called peaks.

Data access links
May remain private before publication.

https://www.ncbi.nlm.nih.gov/geo/query/acc.cgi?acc=GSE143306

Files in database submission

GSM4256783 MZsox19b_K4me3_ChIP
GSM4256784 MZsox19b_K4me3_input
GSM4256785 MZsox19b_K27ac_rep1_ChIP
GSM4256786 MZsox19b_K27ac_rep1_input
GSM4256787 MZsox19b_K27ac_rep2_ChIP
GSM4256788 MZsox19b_K27ac_rep2_input
GSM4256789 MZspg_K4me3_ChIP
GSM4256790 MZspg_K4me3_Input
GSM4256791 MZspg_K27ac_ChIP
GSM4256792 MZspg_K27ac_Input
GSM4256793 WT_K4me3_ChIP
GSM4256794 WT_K4me3_Input

	GSM4256795 WT_K27ac_ChIP<br>GSM4256796 WT_K27ac_Input
Genome browser session<br>(e.g. UCSC)	https://genome.ucsc.edu/s/onichtchouk/danRer11_example1

Methodology

Replicates	GSM4256785 MZsox19b_K27ac_rep1_ChIP<br>GSM4256786 MZsox19b_K27ac_rep1_input<br>GSM4256787 MZsox19b_K27ac_rep2_ChIP<br>GSM4256788 MZsox19b_K27ac_rep2_input<br>Pearson correlation 0.87 (Methods section of the manuscript, sub-chapter "H3K27ac and H3k4me3 ChIP-seq data analysis and visualization"
Sequencing depth	For each sample, ChIP libraries were sequenced to 70 mln reads, Input libraries to 30 mln reads, 2x150bp paired reads. The numbers of total, unmapped, uniquely mapped and non-unique reads are listed in the table below.<br>GEO sample name total paired reads aligned concordantly 0 times aligned concordantly 1 time aligned concordantly >1 times<br>GSM4256783 MZsox19b_K4me3_ChIP 80947078 18732556 39282950 22931572<br>GSM4256784 MZsox19b_K4me3_input 35120106 9792857 15060571 10266678<br>GSM4256785 MZsox19b_K27ac_rep1_ChIP 75997006 19134036 43192163 13670807<br>GSM4256786 MZsox19b_K27ac_rep1_input 27699603 7187932 12287113 8224558<br>GSM4256787 MZsox19b_K27ac_rep2_ChIP 75997006 18772859 34546346 22677801<br>GSM4256788 MZsox19b_K27ac_rep2_input 34639712 10474100 13638635 10526977<br>GSM4256789 MZspg_K4me3_ChIP 57556826 12152715 27764789 17639322<br>GSM4256790 MZspg_K4me3_Input 30025456 5153159 15079853 9792444<br>GSM4256791 MZspg_K27ac_ChIP 83022027 12831469 42493084 27697474<br>GSM4256792 MZspg_K27ac_Input 28948023 5314748 14190612 9442663<br>GSM4256793 WT_K4me3_ChIP 83965733 17376185 40571815 26017733<br>GSM4256794 WT_K4me3_Input 12292749 2401251 5935674 3955824<br>GSM4256795 WT_K27ac_ChIP 85589729 15256390 41705626 28627713<br>GSM4256796 WT_K27ac_Input 19653448 4015882 9110189 6527377
Antibodies	Anti-Histone H3 (acetyl K27) rabbit, 1/100 dilution Abcam plc., Cambridge, UK ab 4729<br>Anti-Histone H3 (tri-methyl K4) rabbit, 1/100 dilution Millipore Co., Temecula, California, USA 07-449
Peak calling parameters	the peaks were called with MACS2 (Feng, J., Liu, T., Qin, B., Zhang, Y. & Liu, X. S. Identifying ChIP-seq enrichment using MACS. Nat Protoc 7, 1728-1740, doi:10.1038/nprot.2012.101 (2012)), input files were pooled and used as a background. The following parameters were used:<br>Effective Genome Size=1370000000, --mfold= 5 to 50, --bw=300, --qvalue=0.05
Data quality	Quality control was performed with FastQC (Andrews, S. (n.d.). FastQC A Quality Control tool for High Throughput Sequence Data. Retrieved from http://www.bioinformatics.babraham.ac.uk/projects/fastqc/) implemented in usegalaxy. eu. The numbers of peaks at FDR 5% and above 5 fold enrichment was more than 100 000 for each ChIP sample
Software	Illumina Casava1.7 software used for basecalling. Data processing was done in usegalaxy.eu public server (Afgan, E. et al. The Galaxy platform for accessible, reproducible and collaborative biomedical analyses: 2016 update. Nucleic Acids Res 44, W3-W10, doi:10.1093/nar/gkw343 (2016)), using Bed Tools (Quinlan, A. R. & Hall, I. M. BEDTools: a flexible suite of utilities for comparing genomic features. Bioinformatics 26, 841-842, doi:10.1093/bioinformatics/btq033 (2010)),<br>Bowtie2 (Langmead, B. & Salzberg, S. L. Fast gapped-read alignment with Bowtie 2. Nat Methods 9, 357-359, doi:10.1038/nmeth.1923 (2012)), DeepTools2 (Ramirez, F. et al. deepTools2: a next generation web server for deep-sequencing data analysis. Nucleic Acids Res 44, W160-165, doi:10.1093/nar/gkw257 (2016)) and MACS2 (Feng, J., Liu, T., Qin, B., Zhang, Y. & Liu, X. S. Identifying ChIP-seq enrichment using MACS. Nat Protoc 7, 1728-1740, doi:10.1038/nprot.2012.101 (2012)).
